# Distinct Structural Determinants of Failure in Morse Taper and Internal Hex Implant Systems

**DOI:** 10.3390/jfb17070346

**Published:** 2026-07-17

**Authors:** Sergio Alexandre Gehrke, Gustavo Coura, Bruno Freitas Mello, Márcio de Carvalho Formiga, Antonio Scarano, Juliana Campos Hasse Fernandes, Gustavo Vicentis Oliveira Fernandes, Fátima de Campos Buzzi

**Affiliations:** 1 Department of Pharmaceutical Science, School of Health Sciences, Universidade do Vale do Itajai, Itajai 88302-901, Brazil; 2 Department of Implantology, Bioface/Postgrados en Odontologia/Universidad Catolica de Murcia, Montevideo 11100, Uruguay; 3 Department of Implantology, Be Dental School, Universidade do Vale do Itajai (UNIVALI), Saint Joseph 88102-700, Brazil; 4 Department of Innovative Technologies in Medicine & Dentistry, University of Chieti-Pescara, 66013 Chieti, Italy; ascarano24@gmail.com; 5 GF10 Foundation, St. Louis, MO 63104, USA; 6 Periodontics, Bitonte College of Dentistry, Northeast Ohio Medical University (NEOMED), Rootstown, OH 44272, USA; 7 Centre for Interdisciplinary Research in Health (CIIS), Universidade Católica Portuguesa, 3504-505 Viseu, Portugal

**Keywords:** dental implants, implant–abutment connection, Morse taper, internal hex, implant diameter, fracture resistance, biomechanical testing, ISO 14801, marginal bone loss, failure mode

## Abstract

**Objectives**: To evaluate the influence of implant–abutment connection design, implant diameter, and simulated marginal bone loss on fracture resistance and failure patterns of dental implant systems. **Materials and Methods**: A total of 180 implant–abutment assemblies were tested, including Morse taper (MT) and internal hex (IH) connections with diameters of 3.5, 4.0, and 5.0 mm. Implants were embedded at two simulated bone levels (0 and 3 mm) and loaded at 30° until failure, in accordance with ISO 14801:2015. Fracture resistance (N) was analyzed using three-way ANOVA. **Results**: Connection type, implant diameter, bone level, and their interactions significantly affected fracture resistance (*p* < 0.001). Internal hex implants showed a marked diameter-dependent increase in resistance and greater reduction under simulated bone loss, particularly in reduced diameters. In contrast, Morse taper implants demonstrated similar resistance values regardless of implant diameter or bone level. Failure patterns also differed between systems: internal hex implants exhibited cervical implant fractures in reduced diameters, whereas Morse taper implants showed progressive abutment deformation and/or abutment fracture without implant body fracture. **Conclusions**: Implant fracture resistance is strongly influenced by implant–abutment connection geometry. Within the limitations of this static in vitro study, MT systems demonstrated diameter-independent mechanical stability and prosthetic-controlled failure patterns, whereas IH systems were highly sensitive to diameter reduction and simulated bone loss.

## 1. Introduction

Dental implants with different diameters have been developed to meet the biomechanical and anatomical demands of distinct clinical scenarios, including variations in interdental space, ridge thickness, emergence profile, and occlusal loading conditions. Traditionally, narrow-diameter implants (NDIs; ≤3.5 mm) were indicated primarily for the replacement of teeth in areas with limited mesiodistal space, such as maxillary lateral incisors and mandibular incisors [1]. Previous clinical studies have demonstrated that NDIs may achieve osseointegration and survival rates comparable to those of regular-diameter implants (RDIs), suggesting that implant diameter alone is not a determinant of biological success [1,2,3,4].

More recently, NDIs have been increasingly used as an alternative to horizontal ridge augmentation procedures in patients presenting reduced alveolar ridge thickness [2,5]. This approach may reduce treatment complexity, morbidity, cost, and surgical time. Nevertheless, the indication for NDIs in posterior regions remains controversial due to biomechanical concerns related to increased occlusal loads and reduced structural resistance [6]. In particular, posterior rehabilitations are commonly associated with higher bending moments, which may increase the risk of mechanical complications such as abutment deformation, screw loosening, or implant fracture [7,8].

Interestingly, recent clinical evidence reports survival rates for NDIs in posterior regions comparable to those observed with regular-diameter implants [9,10]. These findings raise an important biomechanical question about the role of implant diameter in the mechanical behavior of implant-abutment assemblies, especially given that different connection geometries may alter stress distributions and failure pathways.

The mechanical performance of implant–abutment systems is strongly influenced by the geometry of the implant connection [11,12]. Currently, the most commonly used implant connections are hexagonal and Morse taper. Previous studies have shown that these connection designs differ substantially in terms of load transfer, micromovement, stress concentration, and fracture resistance [13,14]. However, it remains unclear whether implant diameter influences the mechanical resistance of all connection systems similarly, or whether distinct connection geometries shift the structural determinant of failure from the implant body to the prosthetic components.

Additionally, marginal bone loss may further increase the bending moment acting on implant–abutment assemblies, particularly in reduced-diameter implants, potentially amplifying the risk of catastrophic mechanical failure [11,12,15]. Therefore, understanding how different implant connections respond to reduced diameter and simulated bone loss conditions may help define safer indications for NDIs in posterior regions with limited bone thickness.

Furthermore, the evolution of implant–abutment connections has led to the development of modern hybrid geometries that attempt to combine the friction-fit sealing capabilities of a deep internal Morse taper with the anti-rotational indexing of an internal hexagon [16]. While classical literature has evaluated purely internal hexagonal or purely conical systems, there is a lack of understanding regarding how fundamental geometric parameters, such as internal taper angles and cervical titanium wall thicknesses, interact with overall fixture diameter to dictate mechanical failure [17]. Under functional loading in the posterior maxilla or mandible, implant assemblies are rarely subjected to pure axial forces; rather, they experience complex, multi-vector off-axis loading that generates significant bending moments across the cervical interface [18]. While static compression testing to failure serves as an indispensable baseline for benchmarking ultimate structural limits and identifying stress concentration bottlenecks, it represents a standardized static simplification of a dynamic oral environment [19]. In vivo, dental implants are subjected to millions of subcritical functional chewing cycles, where progressive metal fatigue, preload loss from embedment relaxation (metal creep), and micro-motion can degrade structural integrity over time [20]. Therefore, evaluating how primary connection designs withstand static off-axis overload under varying bone level support provides vital foundational data that can clarify whether structural failure is dominated by the fixture body or the prosthetic components.

Thus, the aim of this in vitro study was to evaluate the fracture resistance and failure patterns of implant–abutment sets with different connection geometries and implant diameters under two simulated bone levels using a quasi-static loading test. The null hypothesis was that implant diameters would have the same influence on the mechanical resistance of all implant connection systems, regardless of connection design or bone level.

## 2. Materials and Methods

### 2.1. Materials

**Figure 1:**
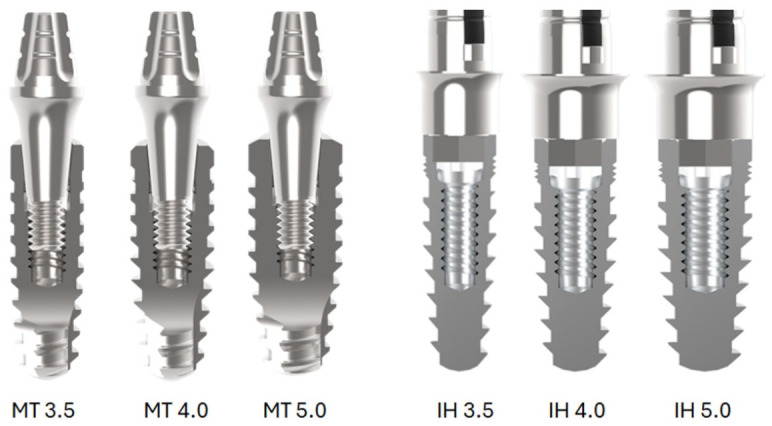
Two implant–abutment connection designs were tested: internal hexagon (IH) and Morse taper (MT). Each connection type was evaluated at three implant diameters (3.5 mm, 4.0 mm, and 5.0 mm) and two bone-level conditions (0 and 3 mm), resulting in 12 experimental groups (n = 15 per group).

A total of 180 implant–abutment (IA) sets were evaluated in the present in vitro study. Implants were manufactured from grade IV titanium, while abutments were manufactured from grade III titanium. Representative images of the tested sets are shown in Figure 1.

To fully characterize the structural mechanics of the evaluated assemblies, precise macrogeometric parameters were standardized across experimental groups. The Morse taper (MT) implant systems featured an internal friction-fit conical connection with an internal total taper angle of 11.5° (5.75° per side relative to the long axis), supplemented by internal threaded sections for abutment screw engagement. The internal hex (IH) implant systems featured a straight hexagonal indexing wall with a depth of 1.5 mm, leading to an apical threaded shaft. The cervical wall thickness of the titanium implant body at the platform interface was directly dependent on the outer diameter of the fixture. For the IH connection assemblies, the cervical wall thicknesses surrounding the hexagonal socket were measured at 0.35 mm (for the 3.5 mm NDI), 0.55 mm (for the 4.0 mm RDI), and 0.95 mm (for the 5.0 mm wide-diameter implant). For the MT conical connection assemblies, the coronal wall thicknesses surrounding the opening of the 11.5° taper were measured at 0.45 mm (for the 3.5 mm diameter), 0.65 mm (for the 4.0 mm diameter), and 1.05 mm (for the 5.0 mm diameter). Both implant systems utilized identical grade IV commercially pure titanium for the implant fixtures and grade III titanium alloy for the prosthetic abutments, ensuring that variations in mechanical behavior were strictly attributable to geometric design rather than metallurgical discrepancies.

### 2.2. Specimen Preparation

**Figure 2:**
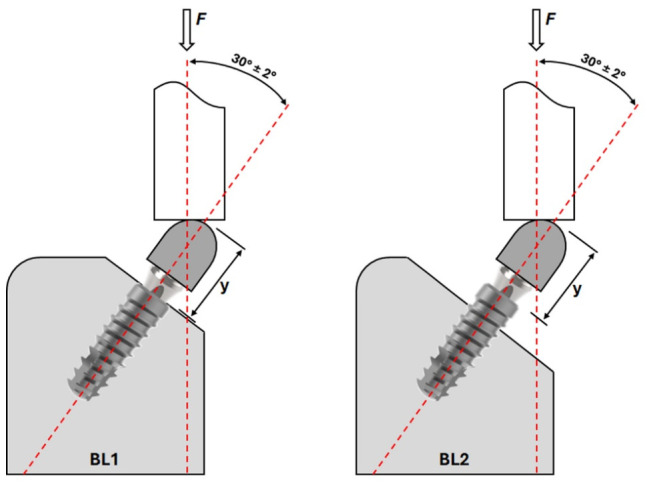
Schematic of the implant positioning with respect to the bone level and the applied load.

All abutments, along with the metallic hemispherical loading caps, had a standardized total height of 8 mm above the implant platform (Figure 2, “y” dimension). Abutments were connected to their respective implants using a calibrated digital torque wrench (Lutron TQ-8800, Lutron Electronic Enterprise Co., Taipei, Taiwan) set to the manufacturer’s recommended tightening torque of 25 Ncm.

To account for the well-documented phenomenon of titanium surface embedment relaxation, commonly known as metal creep, in which microroughness on the mating conical and threaded surfaces yields under the initial clamp load, a standardized retorquing protocol was implemented [21]. Following initial torque application, all implant–abutment assemblies were left to rest for 15 min at room temperature (22 ± 1 °C) to permit stress relaxation and thread settling. Subsequently, each abutment screw was retorqued to the target preload of 25 Ncm prior to cementing the loading caps.

The metallic hemispherical loading caps were fabricated from stainless steel and permanently fixed onto the superior aspect of each abutment using a self-adhesive dual-cured resin cement (RelyX U200, 3M ESPE, Seefeld, Germany) under a constant axial load of 50 N for 10 min, ensuring uniform, point-contact load transfer during mechanical testing. The metallic hemispherical loading cap was fabricated and cemented onto each abutment to standardize load application during mechanical testing.

The implants were embedded in epoxy resin (Polipox Rígida GIV, São Paulo, Brazil), which has an elastic modulus similar to that of human cortical bone. Two different embedding levels were used to simulate distinct marginal bone conditions:

- BL1: Implant platform at the bone level;
- BL2: Implant platform 3 mm out from the bone level, simulated marginal bone loss.

### 2.3. Mechanical Testing

All specimens were subjected to quasi-static compressive loading until failure. Loading was applied at a 30° angle relative to the implant long axis using a universal testing machine equipped with a 5.0 kN load cell (AME-5kN, Técnica Industrial Oswaldo Filizola Ltda., Guarulhos, Brazil). The crosshead speed was set at 1 mm/min. The maximum value until failure (N) was used as the outcome variable for statistical analysis.

The selection of a 30° oblique loading angle relative to the implant longitudinal axis was executed in strict compliance with ISO 14801:2015 [22] specifications. From a biomechanical standpoint, while 0° pure axial compressive loading occurs during centric occlusal clenching and 90° lateral shear vectors can occur during extreme parafunctional bruxism or traumatic impact, neither represents standard functional mastication in the posterior segments. A 30° off-axis vector is internationally standardized because it simulates a severe, worst-case functional bending moment combined with axial compression. This compound loading vector forces the implant–abutment assembly to resist simultaneous compressive, tensile, and shear stresses across the cervical interface, effectively challenging the locking friction of the MT, the rotational walls of the internal hex, and the tensile strength of the retaining screws.

### 2.4. Failure Pattern Analysis

After mechanical testing, fractured specimens were ultrasonically cleaned (Olsen, Palhoça, Brazil) and washed with 96% alcohol. The specimens were then analyzed for characterization of failure patterns and photographic documentation. Failure modes were classified according to the location and type of structural damage, including: (1) abutment deformation, (2) abutment fracture, (3) cervical implant deformation, and (4) cervical implant fracture.

### 2.5. Statistical Analysis

Descriptive statistics, including mean, standard deviation (SD), and 95% confidence intervals (95% CI), were calculated for all experimental groups. Boxplots (minimum, 25th percentile, median, 75th percentile, and maximum) were generated to visualize the distribution of fracture resistance values across conditions.

Inferential analysis was performed using a three-way ANOVA, with the following independent variables: Implant–abutment connection type (MT or IH), implant diameter (3.5, 4.0, and 5.0 mm), and simulated bone levels (0 and 3 mm). Although normality assumptions were violated (Shapiro–Wilk test), the ANOVA was maintained due to the balanced design and confirmation of homogeneity of variances by the Brown–Forsythe test. Post hoc pairwise comparisons were conducted using the Holm–Sidak method, with an overall significance level set at 5%.

Additionally, the percentage reduction in fracture resistance associated with simulated bone loss was calculated for each connection type and implant diameter. A biomechanical risk assessment for reduced-diameter implants (3.5 mm) was also performed using average posterior human masticatory forces reported in the literature. All analyses were conducted using SigmaStat 4.0 (Systat Software Inc., San Jose, CA, USA).

## 3. Results

Descriptive statistics for all experimental groups are presented in Table 1. Given the observed effect sizes and sample size, post hoc power analysis indicated a statistical power ≥ 0.999 (α = 0.05) for all main factors and interaction terms.

**Figure 3:**
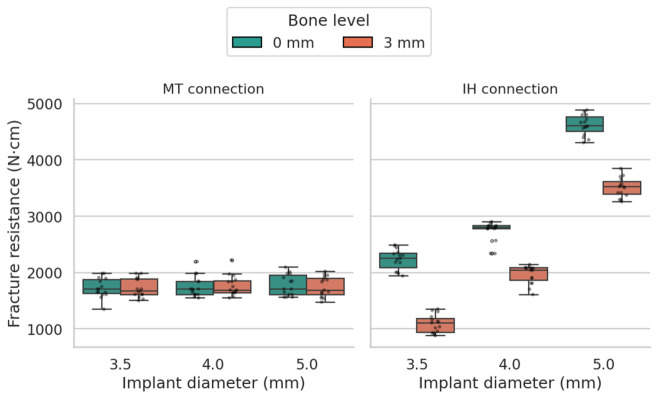
Boxplot distribution of fracture resistance values for Morse taper (MT) and internal hex (IH) implant–abutment assemblies according to implant diameter and simulated bone level. Individual experimental values are superimposed over boxplots. Morse taper implants demonstrated stable resistance regardless of diameter, whereas internal hex implants showed a progressive increase in resistance with larger diameters and a marked reduction after simulated bone loss.

Boxplot distributions for all experimental groups are shown in Figure 3, illustrating variability and central tendency of fracture resistance values across connection types, diameters, and bone levels.

Assumptions of normality were not met according to the Shapiro–Wilk test; however, homogeneity of variances was confirmed by the Brown–Forsythe test. Given the balanced experimental design, a three-way ANOVA was performed considering implant–abutment connection type, implant diameter, and simulated bone level as independent variables.

Biomechanically, the exceptionally high effect size observed for the Connection × Diameter interaction (ηp^2^ = 0.896, F = 721.26, *p* < 0.001) confirms that the structural influence of fixture diameter is fundamentally dictated by the internal connection geometry. In practical terms, this interaction proves that adding outer titanium mass affects the two systems in diametrically opposite ways. For internal hex systems, expanding the diameter from 3.5 mm to 5.0 mm resulted in a massive 106.8% increase in ultimate fracture resistance at the 0 mm bone level (from 2232.5 N to 4618.4 N), because the outer titanium wall surrounding the hexagonal socket directly resists bending forces.

Conversely, for Morse taper systems, expanding the implant diameter across the identical range yielded a statistically insignificant 3.0% increase in fracture resistance (from 1723.1 N to 1775.9 N, *p* = 0.710). This statistical interaction demonstrates that in Morse taper assemblies, the outer fixture diameter is structurally decoupled from the system’s ultimate yield strength under off-axis loading, as the mechanical fuse is entirely isolated within the transmucosal abutment core.

The three-way ANOVA demonstrated significant effects for all main factors and interaction terms (*p* < 0.001), with very large effect sizes observed for implant diameter (ηp^2^ = 0.901) and connection type (ηp^2^ = 0.885), followed by bone level (ηp^2^ = 0.689). Significant interaction effects were also observed, particularly for the connection × diameter interaction (ηp^2^ = 0.896), indicating a strong dependency between implant geometry and connection design (Table 2).

Mean fracture resistance values, reduction (%), and standard deviations for all experimental groups are summarized in Table 3.

No significant differences were observed among implant diameters within the Morse taper groups at either 0 mm or 3 mm bone levels (*p* = 0.710 and *p* = 0.747, respectively). In contrast, internal hex implants exhibited a significant increase in fracture resistance as implant diameter increased (*p* < 0.001).

Simulated marginal bone loss had minimal effect on Morse taper implants, with negligible variation in fracture resistance between bone levels. Conversely, internal hex implants exhibited a marked reduction in fracture resistance following simulated bone loss, particularly in reduced-diameter groups.

**Figure 4:**
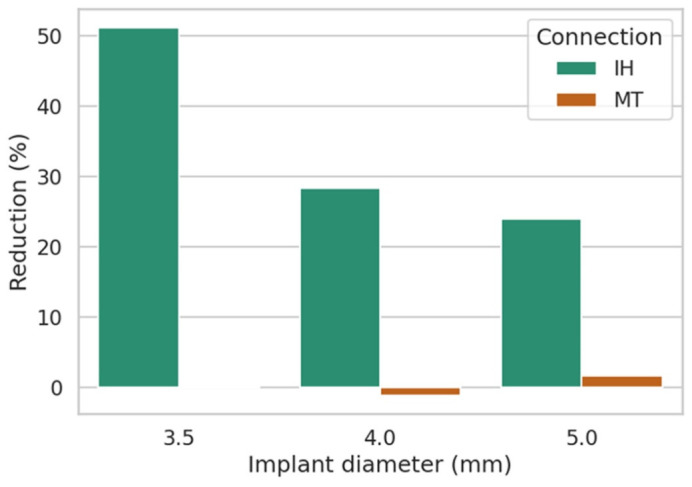
Percentage reduction (%) in ultimate fracture resistance caused by 3 mm of simulated marginal bone loss across connection systems and implant diameters. Internal hex (IH) implants exhibited dramatic, diameter-dependent sensitivity to crestal bone loss, with strength reductions ranging from 23.96% in wide 5.0 mm implants to 51.16% in narrow 3.5 mm fixtures. In contrast, MT implants demonstrated complete biomechanical stability, with negligible variations in fracture resistance (≤1.64%) regardless of simulated bone loss or fixture diameter.

The percentage reduction in fracture resistance associated with bone loss is presented in Figure 4. Internal hex implants exhibited reductions ranging approximately from 24% to 36%, whereas Morse taper implants showed negligible variation between bone levels.

Distinct failure patterns were observed between the evaluated connection systems. In all Morse taper groups, failure was characterized by progressive plastic deformation of the abutment. In the 3.5 mm MT group, deformation was concentrated at the cervical region of the abutment without implant fracture. In the 4.0 mm and 5.0 mm MT groups, deformation progressed until abutment fracture occurred. Importantly, no implant fracture was observed in Morse taper specimens regardless of implant diameter.

In contrast, internal hex implants exhibited implant-dependent failure behavior. In the 3.5 mm and 4.0 mm IH groups, cervical implant fractures were consistently observed. In the 5.0 mm IH group, failure shifted predominantly to the prosthetic component, with fracture occurring at the abutment level.

**Figure 5:**
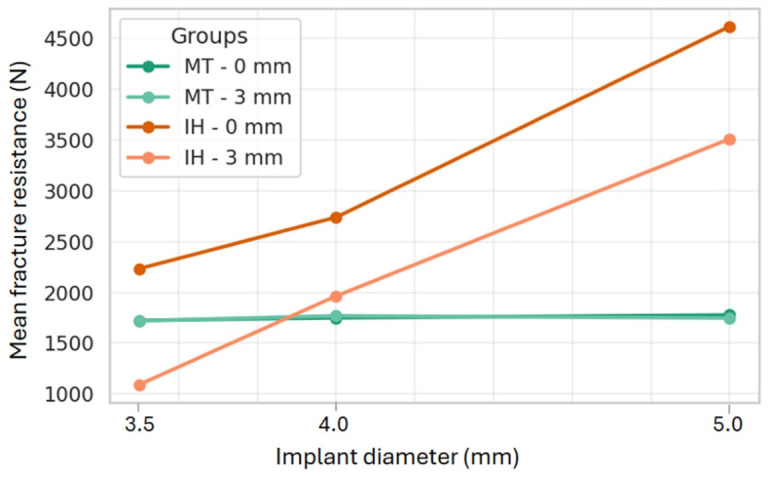
Mean ultimate fracture resistance (N) plotted as a function of implant diameter across 0 mm and 3 mm bone levels. The plot clearly visualizes the divergent mechanical behaviors of the two connection styles: IH assemblies show a steep, diameter-dependent linear increase in strength, whereas MT assemblies maintain a flat, horizontal resistance plateau governed entirely by prosthetic abutment yielding.

These findings indicate that Morse taper systems maintained an abutment-controlled failure pattern independent of implant diameter, whereas internal hex systems exhibited a transition from implant-dominated failure in reduced diameters to prosthetic-component failure in wider diameters (Figure 5).

## 4. Discussion

Mechanical complications involving implant–abutment assemblies remain a primary concern in restorative implantology, particularly in posterior regions subjected to high occlusal forces [23]. Among these, fracture of the implant body is considered a catastrophic failure, often necessitating surgical explantation, extended treatment times, and complex rehabilitation [23,24,25]. While NDIs have historically been associated with an elevated biomechanical risk due to their reduced cross-sectional area [8], the present study demonstrates that mechanical integrity is not solely dictated by diameter; rather, it is fundamentally governed by the interplay between connection design and stress distribution.

The primary finding of this investigation is the stark biomechanical contrast between connection systems. MT implants exhibited robust, diameter-independent fracture resistance, whereas IH implants relied heavily on increased diameter for mechanical stability. Furthermore, MT implants demonstrated a consistent, predictable failure pattern—characterized by progressive abutment deformation—regardless of the implant body diameter. In contrast, narrow-diameter IH implants suffered catastrophic cervical fractures. These divergent outcomes indicate that the connection geometry determines the primary stress concentration locus, thereby dictating the system’s ultimate failure determinant [11,26,27].

In IH systems, fracture resistance is heavily reliant on the cervical wall thickness of the implant body. This is evidenced by the failure transition observed in this study: 3.5 mm and 4.0 mm IH implants failed at the implant neck, while 5.0 mm implants exhibited enough structural rigidity to shift the failure point to the abutment. Conversely, MT implants yielded similar fracture resistance values across all diameters because the transmucosal abutment geometry, the mechanical bottleneck of the system, remained constant. In the MT design, the emergence profile serves as the primary stress concentration zone, protecting the implant body from catastrophic fracture regardless of the implant diameter.

The pronounced statistical interaction between connection design and diameter (ηp^2^ = 0.896) provides objective proof of two distinct mechanical failure mechanisms. In IH implants, the primary resistance to off-axis bending is provided by the outer titanium wall surrounding the hexagonal socket; increasing the implant diameter directly expands this cross-sectional surface area and cervical wall thickness, resulting in dramatic strength gains. Conversely, in Morse MT systems, the solid conical abutment core acts as the mechanical fuse. Because the transmucosal abutment diameter and screw interface remained standardized across the 3.5, 4.0, and 5.0 mm fixtures, structural yielding consistently occurred within the abutment neck before the ultimate stress limit of the outer implant body could be reached.

The introduction of simulated marginal bone loss further highlighted these biomechanical disparities. Crestal bone resorption increases the vertical cantilever effect, amplifying bending moments and stress concentration at the cervical interface [12,28]. In this study, simulated bone loss severely compromised the fracture resistance of IH implants, especially those with reduced diameters. The MT implants, however, remained largely unaffected. This suggests that the deep conical interface of MT systems facilitates a more favorable, apically distributed stress transfer, mitigating the damaging leverage effects of crestal bone loss.

From a clinical standpoint, the localized failure pattern of MT implants serves as a biomechanical failsafe. The ductile yielding and deformation of the abutment prior to fracture dissipates kinetic energy, preventing sudden, catastrophic damage to the fixture itself. Prosthetic failures, while inconvenient, are easily manageable compared to implant body fractures. These in vitro findings provide a strong mechanistic rationale for recent clinical data reporting excellent survival rates for NDIs in posterior regions [9,10]. Recent studies and systematic reviews [9,29,30] have demonstrated that narrow and regular-diameter implants achieve comparable survival and marginal bone stability when supporting single posterior crowns. Our results suggest that when using a Morse taper system, increasing the implant diameter may offer diminishing biomechanical returns, as the system’s strength is limited by the prosthetic component rather than the fixture. Consequently, narrow-diameter MT implants represent a highly viable, minimally invasive alternative in anatomically constrained posterior sites, potentially negating the need for complex ridge augmentation.

### 4.1. Nuanced Mechanical Behavior vs. Absolute Superiority

When interpreting the biomechanical findings of this study, it is critical to emphasize that the stable, diameter-independent fracture resistance observed in MT implants should not be misconstrued as superior absolute mechanical performance. Rather, the data demonstrates two fundamentally different mechanical behaviors governing stress distribution and failure pathways. MT implants demonstrate relatively stable fracture resistance despite reductions in implant diameter or simulated marginal bone loss. IH implants demonstrate greater structural dependence on implant diameter and crestal bone levels.

However, when evaluating absolute load capacity, regular and larger IH implants achieved considerably higher ultimate fracture resistance than MT implants under identical conditions. For example, the 4.0 mm IH implant at the 3 mm simulated bone loss level exhibited a mean fracture resistance of 1963.0 N, which is substantially higher than the 1768.2 N achieved by the 4.0 mm MT implant under the same bone loss condition. Therefore, while MT systems offer predictability and act as a prosthetic failsafe by capping stress within the abutment, regular- and wide-diameter IH systems provide superior ultimate load-bearing capacity before catastrophic failure occurs, provided adequate cervical wall thickness is present.

### 4.2. The Impact of Metal Creep and Preload Maintenance

A vital mechanical factor that influences the fracture resistance and fatigue life of implant–abutment connections is the maintenance of screw preload. In tapered interference fits, such as MT connections, tightening the retaining screw forces the male conical abutment into the female implant sink, generating massive radial clamping forces [31]. However, this high contact pressure induces localized plastic deformation of micro-machining peaks across the titanium mating surfaces, a phenomenon known as embedment relaxation or metal creep [21]. This relaxation inevitably results in a reduction in initial tightening torque and preload within the first several minutes following torque application.

In our experimental protocol, implementing a standardized 15 min resting window followed by retorquing to 25 Ncm was critical to stabilize the conical interface and ensure maximum frictional wedging. In clinical practice, if metal creep is not compensated for by a retorquing protocol, preload loss can lead to micro-motion under oblique masticatory loads. In MT systems, because structural integrity relies heavily on the taper’s friction fit rather than solely on the retaining screw, uncompensated metal creep could prematurely shift bending stresses onto the abutment screw, potentially altering the favorable abutment-yielding failure pattern observed in our static tests [31].

### 4.3. Relevance to Modern Hybrid Implant Connections

In contemporary implant dentistry, manufacturers have increasingly shifted toward “hybrid” connection designs that merge a deep internal conical taper (typically ranging between 11° and 16°) with an apical internal hexagonal or polygonal indexing feature [16,32]. A common clinical and biomechanical question is how the divergent behaviors observed in our purely conical (11.5° MT) and purely hexagonal (IH) systems apply to these modern hybrid macrogeometries.

Based on principles of solid mechanics and our observation of interaction effects, the mechanical behavior of a hybrid connection under oblique functional loading will be governed primarily by which anatomical feature absorbs the majority of the bending moment. When a hybrid implant features a true friction-fit conical taper (≤16°) that is fully seated and properly torqued, the coronal conical interface acts as the primary load-bearing surface [32]. The intimate metal-to-metal contact of the taper absorbs and distributes lateral bending forces across a broad surface area of the implant emergence profile before those forces can reach the apical internal hex.

Consequently, true hybrid connections behave mechanically much like pure MT systems: they exhibit stable, relatively diameter-independent fracture resistance and tend to fail via ductile abutment deformation rather than cervical body fracture. In these hybrid systems, the internal hex serves primarily as a surgical driving tool and a prosthetic anti-rotational indexer during crown placement, rather than a primary resistance center against masticatory bending moments [16].

### 4.4. Limitations and Future Perspectives

The findings of the present investigation must be interpreted strictly within the context of its in vitro design and standardized mechanical testing parameters. While static compression testing to failure in accordance with ISO 14801:2015 provides highly reproducible, comparative benchmarking data regarding ultimate strength (F_max_) and baseline structural bottlenecks, it represents a simplified worst-case scenario that does not fully replicate the physiological complexity of the dynamic oral environment.

First and foremost, the present study evaluated quasi-static loading until catastrophic failure. In clinical reality, dental implants are rarely subjected to a single, acute static overload exceeding 1000 N; rather, they operate under subcritical, multi-directional cyclic loading during mastication and deglutition [19]. The primary cause of clinical implant and prosthetic failure is dynamic cyclic fatigue, in which microscopic cracks initiate at stress-concentration zones (such as screw threads or sharp internal line angles) and slowly propagate through the titanium crystal lattice over millions of cycles [20]. While our static results confirm that MT abutments yield plastically before the implant body fractures under acute overload, cyclic fatigue testing (e.g., loading assemblies through 10^6^ to 5 × 10^6^ cycles at 40% to 80% of their static yield strength) is urgently required to verify whether this abutment-controlled safety mechanism persists under long-term mechanical aging.

Secondly, the experimental embedding model utilizes rigid epoxy resin to simulate human cortical bone. While epoxy resin is standardized by ISO 14801 [22] due to its elastic modulus (~3 GPa), it creates a uniform, rigid, and circumferential support profile around the implant fixture. In vivo, crestal bone resorption is rarely uniform and horizontal; it frequently manifests as irregular, circumferential crater-like defects or angular bony funnels that alter the fulcrum of lateral bending forces. Furthermore, our static model omits the viscoelastic damping properties of surrounding peri-implant mucosal tissues, the cushioning effect of the periodontal ligament of adjacent natural teeth, and the force-dissipating properties of the food bolus, all of which mitigate peak loading stresses in patients.

Finally, this study evaluated a single implant macrogeometry, a standardized 8 mm abutment height above the platform, and specific titanium grades (Grade IV fixtures and Grade III abutments). Extrapolating these results to implants with different thread profiles, shorter or longer prosthetic heights, or alternative metallurgy (such as Grade V Ti-6Al-4V alloy or zirconia ceramics) should be avoided. Future research should combine dynamic cyclic fatigue protocols in wet, temperature-controlled environments (37 °C saline) with advanced three-dimensional Finite Element Analysis (FEA) and prospective randomized clinical trials to map stress concentration gradients and validate the long-term clinical predictability of narrow-diameter tapered connections in posterior sites.

## 5. Conclusions

Within the limitations of this in vitro study, the following conclusions may be drawn: (1) implant diameter significantly influenced fracture resistance in internal hex implant–abutment assemblies, whereas Morse taper systems were not significantly affected by diameter variations; (2) simulated marginal bone loss reduced fracture resistance in internal hex implants, particularly in reduced diameters, while Morse taper implants showed minimal mechanical variation; (3) distinct failure patterns were observed according to connection geometry, with internal hex implants showing cervical implant fractures in reduced diameters and Morse taper implants exhibiting progressive abutment deformation; (4) implant–abutment connection design plays a decisive role in determining fracture resistance and failure mode; (5) within the tested static loading conditions, narrow-diameter MT implants exhibited stable structural resistance comparable to wider diameters However, cyclic fatigue and clinical studies are required before definitive clinical recommendations can be made.

## Figures and Tables

**Table 1:** Descriptive statistics for all experimental groups.

Connection	Diameter (mm)	Bone Level (mm)	Mean (N)	SD	Min	Max
IH	3.5	0	2232.5	181.7	1941	2488
IH	3.5	3	1090.5	158.9	878	1352
IH	4.0	0	2739.1	179.0	2338	2903
IH	4.0	3	1963.0	163.4	1609	2141
IH	5.0	0	4618.4	182.1	4309	4888
IH	5.0	3	3511.8	172.2	3260	3848
MT	3.5	0	1723.1	174.5	1347	1984
MT	3.5	3	1719.7	165.0	1502	1984
MT	4.0	0	1749.1	184.9	1548	2194
MT	4.0	3	1768.2	180.7	1549	2221
MT	5.0	0	1775.9	187.1	1560	2094
MT	5.0	3	1746.9	180.6	1475	2021

IH = internal hexagon; MT = Morse taper; mm = millimeters; N = Newton; SD = standard deviations; Min = minimum; Max = maximum.

**Table 2:** Three-way ANOVA results for fracture resistance (N), including effect size (partial eta squared, ηp^2^).

Source	SS	df	MS	F	*p*-Value	ηp^2^
Connection	4.02 × 10^7^	1	4.02 × 10^7^	1297.79	<0.001	0.885
Diameter	4.72 × 10^7^	2	2.36 × 10^7^	762.08	<0.001	0.901
Bone level	1.15 × 10^7^	1	1.15 × 10^7^	372.32	<0.001	0.689
Connection × Diameter	4.47 × 10^7^	2	2.24 × 10^7^	721.26	<0.001	0.896
Connection × Bone level	1.13 × 10^7^	1	1.13 × 10^7^	365.75	<0.001	0.685
Diameter × Bone level	3.68 × 10^5^	2	1.84 × 10^5^	5.94	0.003	0.066
3-way interaction	2.52 × 10^5^	2	1.26 × 10^5^	4.06	0.019	0.046
Residual	5.21 × 10^6^	168	3.10 × 10^4^	—	—	—

**Table 3:** Mean ultimate fracture resistance values (N) and percentage reduction (%) associated with simulated marginal bone loss across experimental groups.

Connection	Diameter (mm)	0 mm (Mean N)	3 mm (Mean N)	Reduction (%)
IH	3.5	2232.47	1090.47	51.16
IH	4.0	2739.13	1963.00	28.33
IH	5.0	4618.40	3511.80	23.96
MT	3.5	1723.13	1719.67	0.20
MT	4.0	1749.13	1768.20	−1.09
MT	5.0	1775.93	1746.87	1.64

## Data Availability

All data generated or analyzed during this study are included in this published article.
